# Spatially Varying Selection Amplifies Intrapopulation Differentiation Among Phenotypic Traits in the Rocky‐Shore Mussel, *Mytilus californianus*

**DOI:** 10.1002/ece3.71641

**Published:** 2025-06-30

**Authors:** Casey S. Richards, Diana Vera Cruz, Jason W. Shapiro, J. Timothy Wootton, Catherine A. Pfister

**Affiliations:** ^1^ Department of Ecology & Evolution University of Chicago Chicago Illinois USA; ^2^ Center for Research Informatics University of Chicago Chicago Illinois USA

**Keywords:** divergent selection, environmental gradients, local adaptation, phenotypic plasticity

## Abstract

Strong ecological gradients along heterogeneous environments play an important role in shaping population differentiation across species ranges. Thus, the selective pressure of environmental variation on phenotypic variation strongly affects an organism's ability to persist across diverse or new environments. We investigated the spatial variability of biological responses in the intertidal bivalve *Mytilus californianus* to highlight the costs and trade‐offs of local adaptation and phenotypic plasticity across various functional traits in a dynamic environment, the marine intertidal. To test this, we performed a reciprocal transplant experiment with *M. californianus* individuals originating from the upper and lower intertidal measuring relevant phenotypic traits, followed by whole genome sequencing (WGS). We determined that morphological traits in individuals demonstrated phenotypic plasticity when moved to new environments, whereas physiological traits such as metabolism exhibited constraints in plasticity. Additionally, mussels from high intertidal zones, which experience greater heat and aerial exposure stress, maintained lower metabolic rates and showed increased frequencies of non‐synonymous mutations in functionally relevant heat shock proteins when compared to low intertidal mussels. These results suggest that morphological and physiological traits responded differently to spatially varying selection within the marine intertidal.

## Graphical abstract

Strong ecological gradients along heterogeneous environments play an important role in shaping population differentiation across species ranges. We investigated the spatial variability of biological responses in the intertidal bivalve *Mytilus californianus* to highlight the costs and trade‐offs of local adaptation and phenotypic plasticity across various functional traits in a dynamic environment, the marine intertidal. We determined that morphological traits in individuals demonstrated phenotypic plasticity when moved to new environments, whereas physiological traits such as metabolism exhibited constraints in plasticity.

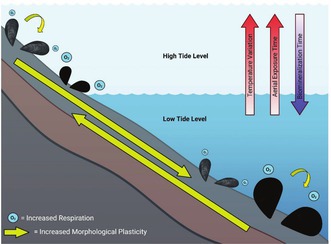

## 1 Introduction

Understanding how species adapt to their environments is a fundamental theme in biology. Strong ecological gradients along heterogenous environments reveal the patterns and processes that shape population genetic structure and phenotypic variation across species ranges (Endler 1986). Across heterogenous environments the interplay between environmental and genetic differences on phenotypic variation can result in the local adaptation of populations (Levins 1968), where local genotypes have higher fitness in their indigenous environment compared to immigrating genotypes from neighboring populations (Savolainen et al. 2013). For connected populations, gene flow prevents the evolution of local adaptation when the strength of selection cannot overcome the stabilizing effects of migration (Wright 1969) and limits adaptive genetic differentiation over steep environmental gradients (Bachmann et al. 2020; Lenormand 2002). Therefore, over microgeographic scales selection may favor “phenotypically plastic phenotypes” opposed to “fixed phenotypes” that have increased fitness in a particular environment (Levins 1968; Scheiner 1998; Hollander 2008). For example, phenotypic flexibility are reversible phenotypic transformations that allow individuals to respond to environmental stochasticity over time, while developmental plasticity allows individuals to respond to heterogeneous environments across space (Piersma and Drent 2003). However, there is growing evidence that local adaptation occurs over microgeographic scales both in terrestrial systems (e.g., Antonovics 2006; Yadav et al. 2021) and especially in marine systems (e.g., Thomas et al. 2022; Gurski 2016; Sanford and Kelly 2011; Hays et al. 2021), suggesting strong selection associated with spatial environmental heterogeneity could drive local adaptation even with population connectivity (Yeaman 2022; Yeaman and Otto 2011).

Coastal marine systems are dynamic, and the intertidal zone is especially so, with many species residing in environments where tides ebb and flow daily, imparting strong changes in temperature, dissolved oxygen (DO), and pH. The variety of responses intertidal species exhibit from living in a dynamic environment has likely contributed to the intertidal being one of the most biodiverse places on the planet. Rocky shores along the marine intertidal are no exception, as they exhibit high biodiversity and experience large variations in temperature, pH, DO, and nutrient supply on seasonal and daily time scales. As a result, rocky shores have attracted much attention over the years for experimental analysis of population and community structure in response to spatial elevation, as temperature variation, sunlight, and aerial exposure increase with height (Paine 1974; Wootton 2005; Connell 1972; Dayton 1971). These extreme environmental stress gradients in the marine intertidal over space and time can select phenotypic traits important for survival. Testing species plasticity and resilience across steep environmental gradients on small spatial scales in the rocky intertidal provides insight into the phenotypic response to stressors, the possibility for adaptation, and the role of genetic variation in shaping the evolution of populations that persist throughout heterogeneous environments.

In marine systems, most organisms reproduce via broadcast spawning, potentially connecting populations over large distances and possibly limiting adaptive differentiation. Yet many broadcast spawning species exhibit spatially structured genetic variation within populations (e.g., Thomas et al. 2022; Palumbi 1994; Luttikhuizen et al. 2003). Foundational species that broadcast spawn are particularly interesting for studying local adaptation because they experience high population connectivity across rapidly fluctuating ocean environments, are numerically abundant, and significantly affect ecosystem function (Ellison 2019; Dayton 1972). *Mytilus californianus* , a broadcast spawning bivalve that dominates rocky intertidal shores of the northeast Pacific Ocean, serves as a foundational species by forming habitats and providing food for a myriad of species (Gutiérrez et al. 2003; Paine and Levin 1981). *M. californianus* experiences strong environmental gradients over relatively small spatial scales and may show high intrapopulation diversity as a result (Hays et al. 2021). Previous analysis of gene expression of in situ *M. californianus* mussels originating from the high intertidal showed a reduction in expression patterns of genes involved in metabolic activity and perturbations to cellular homeostasis when compared to in situ low intertidal mussels (Place et al. 2012). How the environment determines expression patterns in *M. californianus* mussels is unknown and could be the result of phenotypic plasticity or local adaptation. The induction of genes involved in stress response for intertidal mussels may come at a substantial energetic cost to other biological functions, specifically a trade‐off between increased fitness and growth rate (Lang et al. 2009). Untangling genetic versus phenotypic responses on spatially varying individuals provides insight into the scope of mussel response across diverse environments on microgeographic scales.

In this study, we assessed functional traits within a continuous mussel population on a steep sloping shelf to test the role of strong environmental differences on phenotypic plasticity versus genetic response. To achieve this, we used reciprocal transplant experiments combined with next generation sequencing to identify intrapopulation genetic and phenotypic variation between *M. californianus* individuals positioned in the low and high intertidal. This study addresses three questions:

1. Are observed phenotypic differences between low and high *M. californianus* individuals a result of phenotypic plasticity or another mechanism such as local adaptation or canalization?
2. If there is evidence of local adaptation based on signatures of selection in candidate Single Nucleotide Variants (SNVs)?
3. What are the costs and trade‐offs between environment and indigenous locale on important phenotypic traits in *M. californianus* individuals on microgeographic scales?

Given that experimental mussels were in close proximity and a part of a continuous mussel bed, the stabilizing effects of high gene flow (Wright 1969) combined with high panmixia observed in many broadcast spawning species (Lourenço et al. 2017; Tay et al. 2016; Ridgway et al. 2001) will limit adaptive genetic differentiation over steep environmental gradients (Bachmann et al. 2020; Lenormand 2002). Therefore, we hypothesized that selection would favor phenotypic plasticity over local adaption (Levins 1968; Scheiner 1998; Hollander 2008). Because in situ *M. californianus* mussels originating from the high intertidal show a reduction in the expression of genes involved in metabolic activity (Place et al. 2012), we hypothesized reduced metabolic activity for mussels exposed to the more stressful high intertidal would trade‐off with increased survival, resulting in a cost to growth (Lang et al. 2009). Testing species plasticity and resilience across steep environmental gradients on small spatial scales in the rocky intertidal provides insight into the phenotypic response to stressors, the possibility for adaptation, and the role of genetic variation in shaping the evolution of populations that persist throughout heterogeneous environments.

## 2 Methods

### 2.1 Reciprocal Transplant Experiment

**FIGURE 1:**
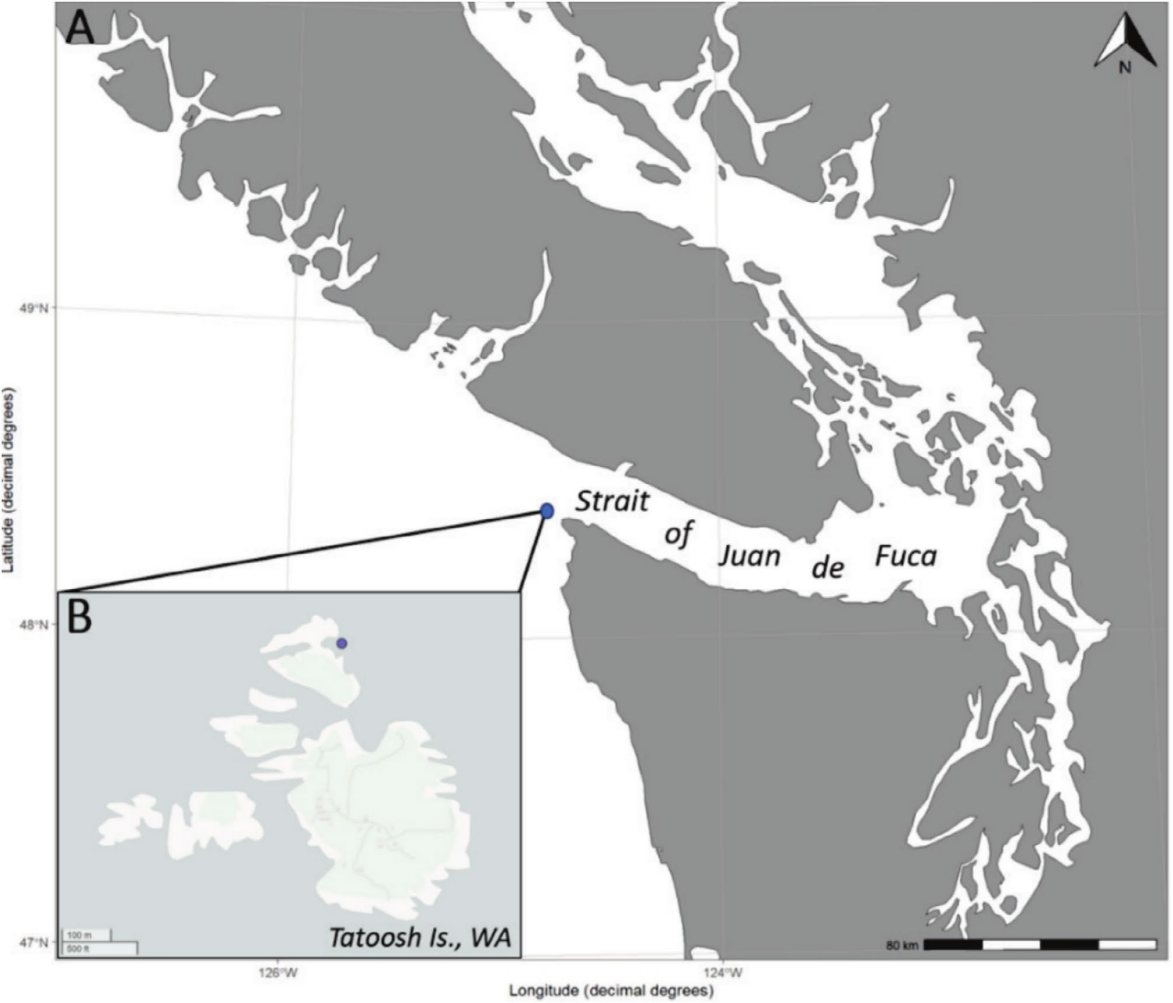
(A) Map of Strait of Juan de Fuca region. (B) Map of Tatoosh Island, Makah Indian Reservation, WA. Purple dots indicate location of Tatoosh Island (A) and site of experimental population and reciprocal transplant experiment (B).

**FIGURE 2:**
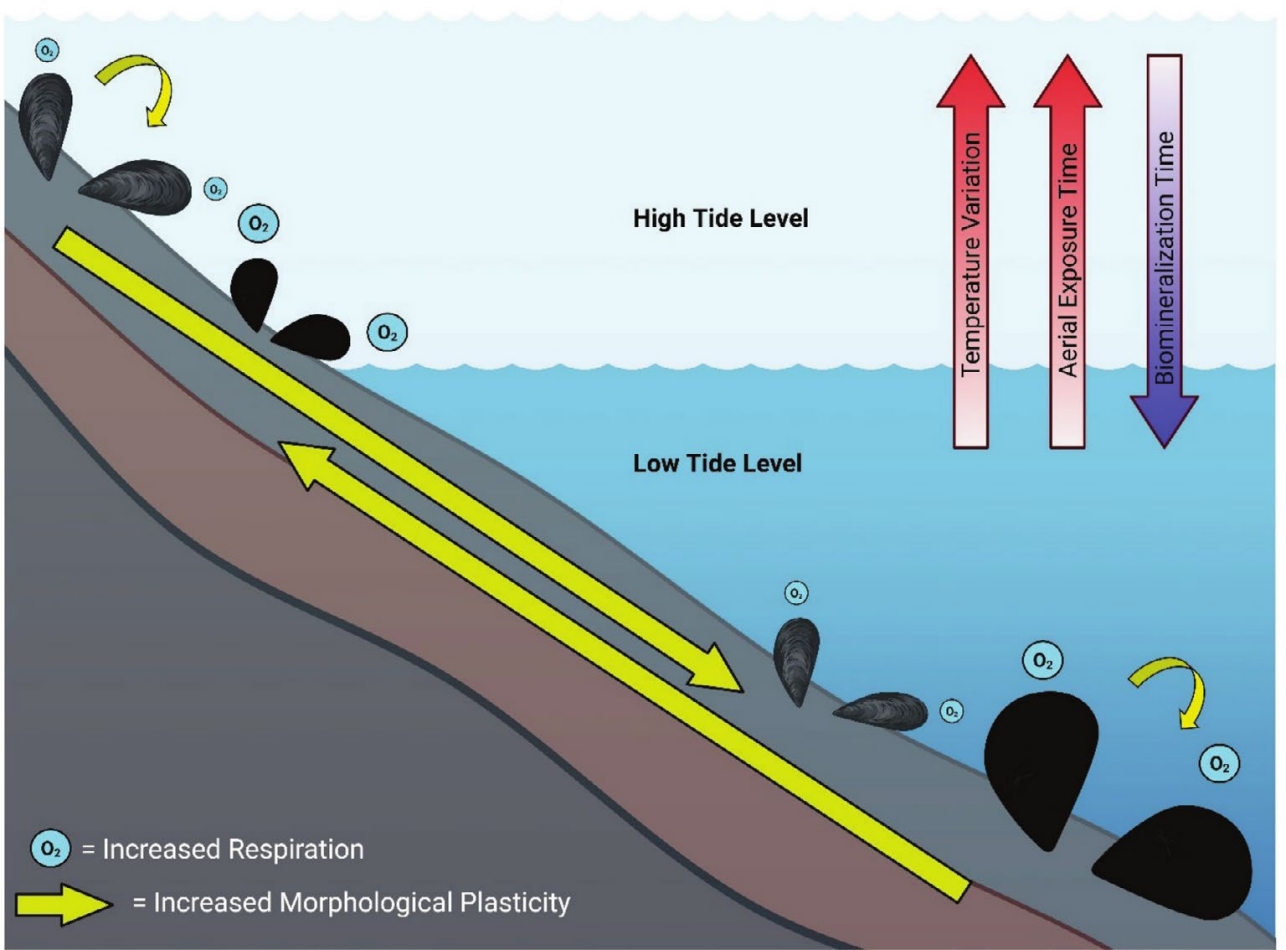
Graphical summary of the results from a reciprocal transplant experiment between *Mytilus californianus* individuals indigenous to the upper (gray mussels) and lower (black mussels) intertidal. Yellow arrows denote the reciprocal transplant experiment, with arrow thickness representing the degree of plasticity. Gray shells of mussels indigenous to the high intertidal represent loss of periostracum from increased incidence of endolithic phototrophs.

**FIGURE A1:**
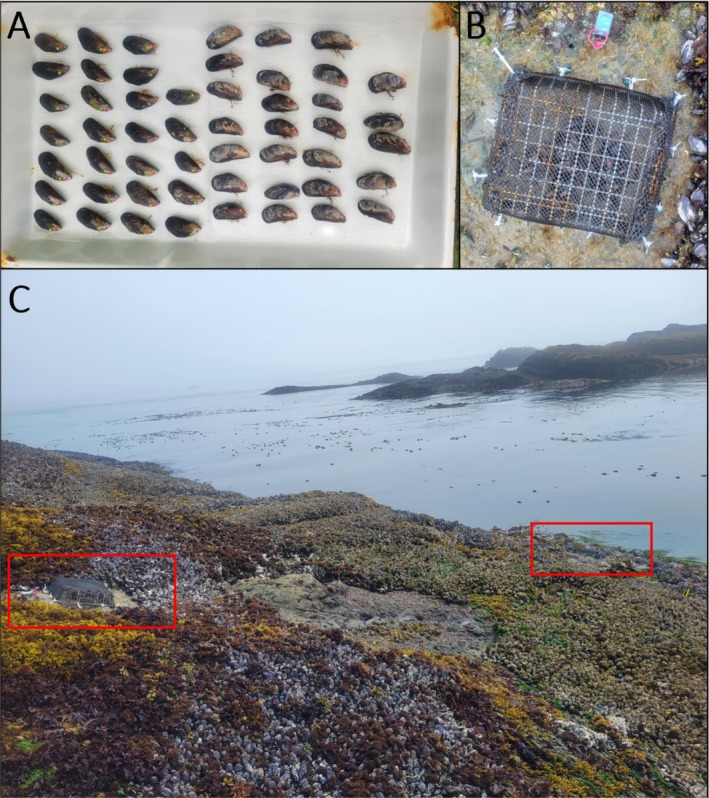
Site and design of reciprocal transplant experiment. (A) Individual mussels marked using glued bee tags and EZ Poxy paint on umbo of left and right shell valve (not all individuals depicted). (B) Cages with vexar mesh to house individual mussels in experimental plots. (C) Site of experimental intertidal plots for in situ and transplanted mussels.

The experimental tractability of mussels allowed a test of how plastic or fixed mussel responses were to intertidal stressors. Individual mussels that were naturally growing in either the low and high intertidal, ranging from 39 to 51 mm in length, were collected during a −0.41 m low tide in 2022 across a steep sloping shelf along a continuous mussel bed on northern Tatoosh Island, Makah Indian Reservation, WA (48°23.680′ N, 124°44.174′ W) (Figure 1A,B). Immediately following collection, individual mussels were transferred into fresh seawater. Initial measurements on all individuals included maximum shell length, shell width, shell height, total weight in air, and buoyant weight as a proxy and non‐destructive method for obtaining approximate shell weight (Palmer 1982) using calipers and an Ohaus precision balance. All individuals were marked on the umbo of the left or right shell valve to designate indigenous locale using bee tags adhered with J‐B KWIK Weld and “Fire Red” EZPoxy paint (PETTIT) (Figures 1, A1 in Appendix A) to characterize individual mussels throughout the reciprocal transplant experiment. Individually labeled mussels were then randomly selected and evenly distributed by treatment: either transplanted back to their original intertidal position (in situ) or cross‐transplanted to the experimental tidal position in the low or high intertidal where they had not originally been living (transplant) (Figure 2). Mussels were housed in two separate 19 L buckets based on assigned treatment plot with fresh seawater maintained at 11°C overnight until individuals could be placed in their respective plot during the following morning low tide. This overnight period also allowed for in situ and transplanted individuals going into the same treatment plot to attach to each other through byssus production, which formed a large cluster of mussels that increased the likelihood for survival when placed under their experimental enclosures in the field (personal observation). The experimental enclosures for both intertidal plots consisted of a steel cage wrapped with 1/4″ plastic Vexar mesh held together with zip ties (Figure A1B in Appendix A). Experimental enclosures were zip tied to stainless steel screw eyes anchored to the rocks in the low and high intertidal plots (Figure A1C in Appendix A). In total, 44 mussels were placed in each plot in the intertidal (88 mussels total), where each treatment plot consisted of 22 in situ and 22 transplanted individuals.

**FIGURE A2:**
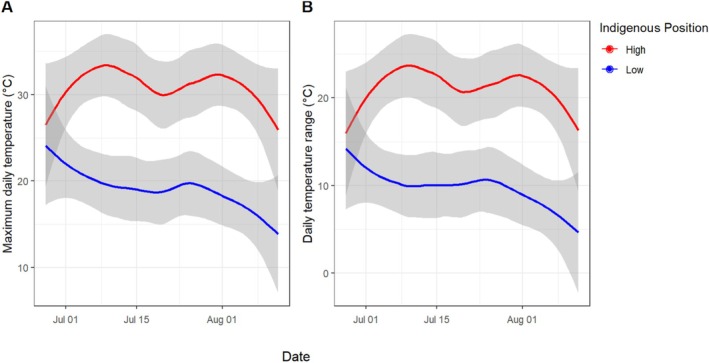
Regression lines of (A) maximum daily temperatures and (B) daily temperature ranges of low and high intertidal positions recorded in 5‐min intervals with Onset HOBO data loggers. Red and blue regression lines are from actual data using locally estimated scatterplot smoothing (LOESS) line where shaded gray areas represent the 95% CI.

The reciprocal‐transplant experiment was initiated in June 2022 and lasted 44 days. The hottest air temperatures occur during the summer months of the year, but were intensified during the study by a record setting heat wave in July 2022 in the Pacific Northeast (NOAA National Centers for Environmental Information 2022) resulting in the high intertidal plot experiencing significantly hotter than ‘normal’ aerial exposure temperatures for prolonged periods when low tides occurred during daytime periods (see results and Figure A2 in Appendix A). These intense stressors resulted in 100% mortality for all mussels positioned in the high intertidal plot between days 18 and 29. Therefore, on day 30 we repeated the methods above to replicate another high intertidal plot consisting of 10 in situ and 10 transplanted mussels that were placed under the experimental enclosure on the morning of day 31. Six of these individuals did not survive (three in situ, three transplants) over the course of the next 13 days, bringing the sample size to seven in situ and seven transplanted mussels for the high intertidal plot. In contrast, 43 out of 44 individuals survived in the low intertidal plot throughout the 44‐day experiment. At the conclusion of the experiment the enclosures were removed, and mussels were collected and kept in fresh seawater maintained at 11°C until final measurements could be obtained. We recorded maximum shell length, shell width, shell height, total weight in air, buoyant weight, and respiration rates for each individual in each treatment. After these measurements were obtained, a tissue sample of the gill was collected for DNA comparison between experimental mussels who originated from the low and high intertidal. The shells of the individual mussels were dried for 72 h at 50°C in a drying oven to obtain dried shell weight. After this the left shell valves were coated with J‐B KWIK Weld, sliced longitudinally (umbo–ventral margin) with a trim saw equipped with a diamond saw blade, and aged under a dissecting scope following the methods from McCoy, Kamenos (McCoy et al. 2018).

To test whether mussel size was important to individual plasticity, a second transplant experiment was performed in May 2023 with smaller “juvenile” individuals that were half the length of mussels in the first experiment (12–28 mm). Both the low and high intertidal plots contained 40 individuals (80 total across the entire experiment) at the beginning of the experiment. The methods and experimental site were the same as the reciprocal transplant but the experiment used only juvenile mussels living in the low intertidal and lasted 74 days to increase the chance of detecting plasticity. Juvenile mussels were not sourced from the high intertidal because they could not be differentiated from slow growing adults nor other *Mytilus* spp. who dominate the high intertidal (Suchanek 1978). Throughout the experiment, only four of these 80 individuals perished, all of them individuals transplanted to the high intertidal plot. Unlike the reciprocal transplant experiment performed with adult mussels, tissue samples, dried shell weight, and age data were not collected on these juveniles as they were returned to their respective treatment plots for an additional 333 days where morphometric and tissue weight data were recorded using the methods described below. Of the 76 individuals returned to the intertidal, 55 survived.

### 2.2 Temperature and Elevation Measurements

Vertical elevation of each plot was determined by calculating meters (m) above mean lower low water (MLLW) using preexisting markers on Tatoosh Island from Kandur (Kandur 2014) and a DEWALT Laser Level with Tripod. In this study, mussels were sampled from sites at 1.10 m (low intertidal) and 2.37 m (high intertidal) above MLLW. Temperature measurements were recorded in 5‐min intervals throughout the entire experimental period for both intertidal plots using Onset HOBO data loggers that were attached to anchored screw eyes positioned next to the experimental enclosures (Figure A1B in Appendix A).

### 2.3 Morphological Trait Measurements

To test whether each treatment had an effect on the phenotypes of individual mussels, changes in shell length (mm), shell width (mm), shell height (mm), shell weight (g), tissue weight (g), and total weight in air (g) were recorded for all individuals used in the reciprocal transplant experiment. These changes in morphological traits for each individual were determined by calculating the differences between measurements at the beginning and the end of the experiment divided by the number of days in their experimental plot. Shell and tissue weight changes were estimated because precise shell weights could only be obtained at the conclusion of the experiment once mussels had been sacrificed. To achieve these estimates, we derived a least squares linear regression model using the dried shell weight (*Y*) and final buoyant weight (*X*) of each individual mussel, which explained > 99% of the variation (*R* ^2^ = 0.995), then estimated the beginning shell weight for each individual using the beginning buoyant weight. Because tissue is neutrally buoyant when submerged (Palmer 1982), we subtracted total weight in air from shell weight to obtain estimated tissue weights at the beginning and end of the experiment.

### 2.4 Respiration Rate

Mussel respiration rates were quantified at the conclusion of the transplant experiments after final morphological measurements had been collected. Mussels were randomly selected from each treatment and kept in fresh seawater maintained at 11°C for approximately 1 h prior to measuring respiration rates from oxygen consumption (mg O_2_ g^−1^ h^−1^). This was achieved by placing each mussel in their own individual closed 12‐oz glass container filled with fresh seawater and DO was measured every second over a 1 h period using a FireSting FSPRO‐4 equipped with oxygen sensor spots and a temperature sensor (Pyroscience, Aachen, Germany). All containers were maintained at 11°C and kept in darkness. Seawater controls lacked mussels but had the same freshly collected seawater used with mussels. Metabolic rates from an additional 32 mussels from a neighboring population (l6 in situ low individuals, l6 in situ high individuals) that were not used in the reciprocal transplant experiment were assayed as an additional control to quantify if the transplantation itself had a significant effect on mussel function.

### 2.5 Net Calcification Rate and Growth Rate

The net calcification rates (g CaCO_3_ day^−1^) for all individuals used in the reciprocal transplant experiment were estimated from changes in their buoyant weight (g) throughout the experiment divided by the number of days in their experimental plot, following the methods of Duarte, Navarro (Duarte et al. 2015). For the transplant experiment with juvenile mussels, net calcification totals (g CaCO_3_) were recorded by subtracting the final buoyant weight for each individual from the beginning average buoyant weight. Growth rates were determined by calculating the differences in shell length (mm) at the beginning and the end of the experiment divided by the number of days in their experimental plot.

### 2.6 Intrapopulation Genetic Diversity

**FIGURE A3:**
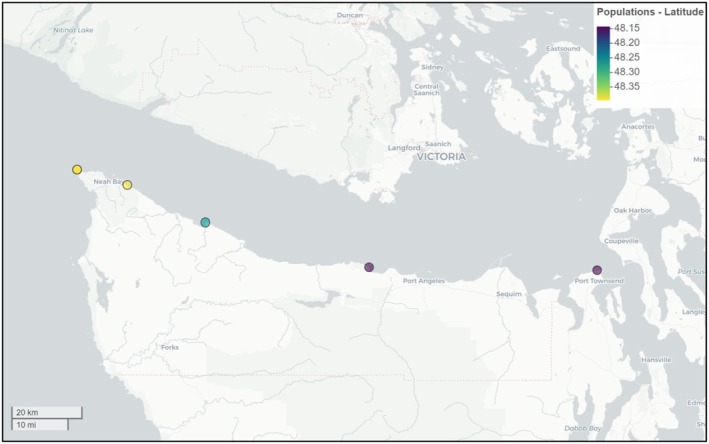
Locations of sampling sites 148 km along the Strait of Juan de Fuca where 54 *Mytilus californianus* individuals were collected for whole genome sequencing. Locations from left to right: Tatoosh Is., WA 48.394667, −124.736233; Snow Creek Boat Launch, WA: 48.355883, −124.547683; Slip Point, WA: 48.263817, −124.251717; Freshwater Bay, WA: 48.150733, −123.637883; Port Townsend, WA: 48.14415, −122.77735.

After all biological measurements were recorded, gill tissue from 10 individuals used in the experiment, five indigenous to the low intertidal and five indigenous to the high intertidal, was collected and immediately placed in RNAlater (Invitrogen) and stored at −20°C until DNA extractions could be completed. DNA was extracted from gill tissue using the E.Z.N.A. DNA/RNA Isolation Kit (Omega Bio‐Tek) following the manufacturers protocols. To obtain regional genomic data and identify candidate Single Nucleotide Variants (SNVs) for the experimental population, an additional 44 *M. californianus* mussels were collected across four populations extending 148 km eastward into the Strait of Juan de Fuca (Figure A3 in Appendix A). All 54 extracted DNA samples were sent to the University of Chicago Functional Genomics Core Facility (RRID:SCR_019196) where whole genome DNA libraries were constructed and sequenced on two NovaX‐10B‐300 lanes with an average coverage of 8.3× per sample.

### 2.7 Germline Variant Calling and Annotation

Germline variant calling and annotation were conducted using various bioinformatics tools for read preprocessing, alignment, variant calling, filtering, and annotation using the *M. californianus* assembly (NCBI RefSeq assembly: GCA_021869535.1) as a reference genome. Data were processed using the nextflow pipeline nf‐core/sarek v3.1.2 (10.12688/f1000research.16665.2, 10.5281/zenodo.4063683). The sarek pipeline performed read preprocessing using FastP (0.23.2) and alignment using BWA‐mem (0.7.17). BAM files were processed using the MarkDuplicates, BaseRecalibrator, and ApplyBQSR tools, followed by variant calling using HaplotypeCaller from the GATK suite version 4.2.6.1. The resulting variants per sample were further filtered and consolidated with the GATK SelectVariants, GenomicsDBImport, GenotypeGVCFs tools, and Picards's MergeVCFs. The filter criteria aimed to keep germline variants with the PASS filter tag. Variant annotation was performed using snpEff toolbox (Cingolani et al. 2012).

Initial analysis yielded over one million variants per sample, which was a result of a large number of duplicated genes within the *M. californianus* reference genome. To overcome this issue, we used a reciprocal BLAST search to identify highly similar regions within the reference genome. These included regions that were over 95% identical or with an alignment length greater than 300 nucleotides. The remaining CDS regions in the genome were added to a BED file and used to filter the variant calling results to recover the most reliable SNVs. EggNOG‐mapper 2.1.12 was used to provide additional functional annotations of candidate SNVs.

### 2.8 Variant Filtering and Analysis

**FIGURE A4:**
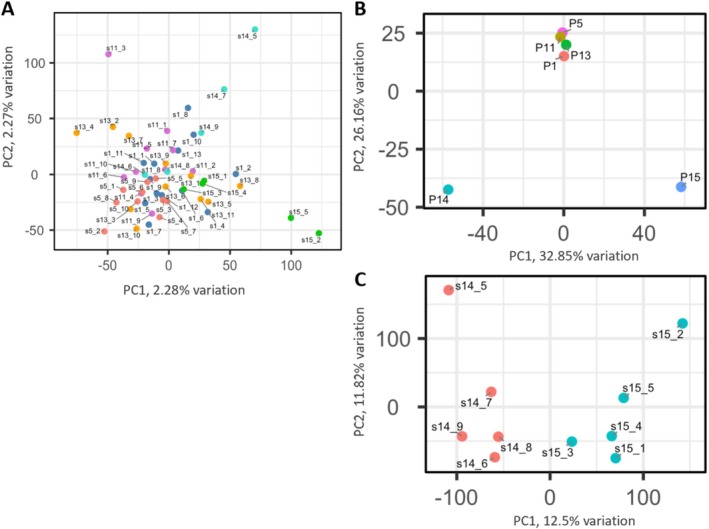
(A) Principal component analysis (PCA) of variant frequency for 54 *Mytilus californianus* individuals and (B) five populations using 133,617 single nucleotide variants that had coverage in all samples and were present in at least one population at a frequency of 50% or higher. Numbers s14 (low intertidal) and s15 (high intertidal) in (A) correspond to Tatoosh Is. individuals. P14 (low intertidal) and P15 (high intertidal) in (B) correspond to the Tatoosh Is. population. (C) PCA using 19,000 single nucleotide variants that had an absolute difference of proportion larger or equal to 0.0075 between 10 low (red dots) and high (blue dots) intertidal *M. californianus* individuals used in a reciprocal transplant experiment.

Annotated, biallelic SNPs with the PASS filter tag and complete GT tag were used for the subsequent analysis using R. A total of 133,617 variants with coverage in all the samples, defined genotypes, and present in at least 1 population at a frequency of 50% or higher were used to create a genotypes matrix and a matrix variant frequency per population. To identify variants that distinguish populations, PCA was calculated using the genotypes matrix for individuals (Figure A4A in Appendix A) and variant frequency per population matrix (Figure A4B in Appendix A). The number of SNVs chosen for a given PC was proportional to the explained variation related to that PC. For each variant, the *F*_st_ coefficient was calculated using the genotypes of all the samples per population. For comparison between low and high individuals sourced from the experimental population, only the top variants that had significant loadings above 0.0075 or below −0.0075 for PC1 were used since PC1 distinguished individuals by their population in an agnostic PCA based on the sample's genotypes (Figure A4C in Appendix A). This process identified 638 SNVs which were used as our candidate SNVs in our population analysis.

### 2.9 Statistical Analyses

All analyses were conducted using the R statistical platform version 4.4.1 (R Core Team 2024). A one‐way ANOVA was used to test for differences in temperature between the two intertidal plots, morphological characteristics (beginning total weight, shell height, shell weight, shell width), physiological characteristics (net calcification rate, respiration rate), and tissue weights of juvenile mussels used in the long‐term transplant experiment. All biological responses (shell height change, shell weight change, total weight change, shell width change, growth rate, respiration rate, net calcification rate, tissue weight) were analyzed using a two‐way ANOVA, with indigenous locale and treatment (in situ or transplant) as the main fixed factors. Differences between groups (a posteriori comparison) were evaluated using a Tukey HSD test. We used a Breusch–Pagan test to test for heteroscedasticity. When data were heteroscedastic, log transformation did not alter overall significance conclusions for any ANOVA. Principal component analyses (PCA) were used to capture phenotypic variation among treatments and intrapopulation genetic variation and SNVs discovery between individuals who were indigenous to the low or high intertidal (Horne and Camp 2004). Phenotypic variation among treatments was also tested using permutational multivariate analysis of variance (PERMANOVA) with the *adonis2* function in *vegan* (999 permutations) (Oksanen et al. 2018), based on Euclidean distances. Z‐standardization was performed on phenotypic trait data prior to PCA and PERMANOVA. The Mann–Whitney *U* test was used to compare metabolic rates of experimental and non‐experimental individuals of differing sample sizes. An *F*‐test for homogeneity of variance was used to compare age variability differences between mussels indigenous to the low and high intertidal.

## 3 Results

### 3.1 Contrasting Temperatures Between Spatially Varying Intertidal Positions

The low and high intertidal demonstrated contrasting environmental conditions, where the low intertidal had a mean temperature of 11.3°C and the high intertidal had a significantly greater mean temperature of 15.6°C (one‐way ANOVA: *F*_(1,26,471)_ = 4851, *p* < 0.0001), indicating the longer aerial exposure times for high intertidal individuals. The average daily range of temperature was two times greater in the high intertidal compared to the low (21.5°C vs. 9.6°C). The low intertidal reached a maximum air temperature of 32.8°C; the high intertidal exceeded this temperature on 20 of the 44 days the temperature logger was deployed, reaching a maximum air temperature of 43.4°C (Figure A2 in Appendix A).

### 3.2 Phenotypic Traits Differ With Tide Height

**FIGURE 3:**
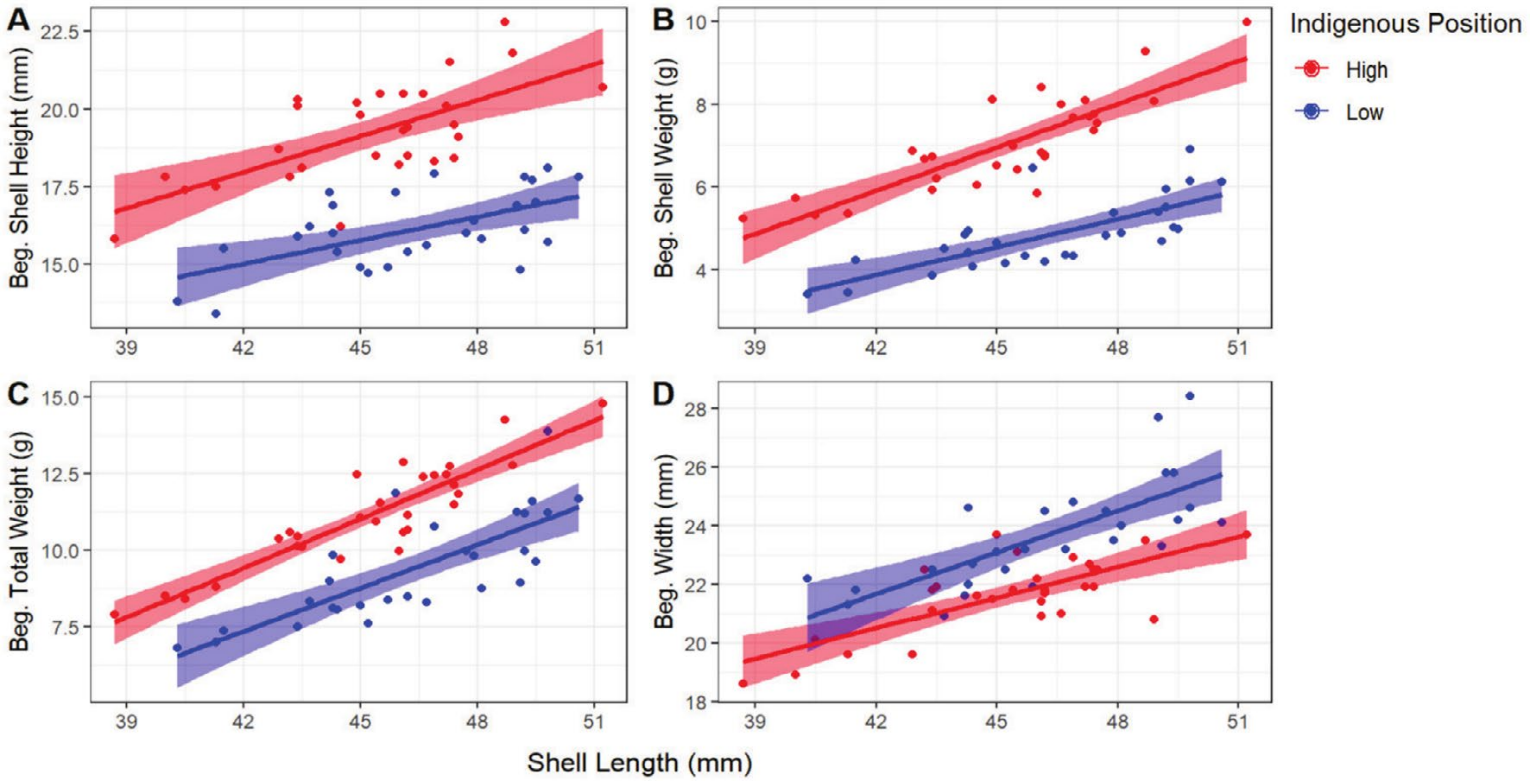
Relationship between maximum shell length (mm) and (A) shell height (mm), (B) shell weight estimated as the buoyant weight (g), (C) total weight measured as weight in air (g), and (D) shell width (mm) of low and high intertidal adult (38–51 mm) mussels at the beginning of a reciprocal transplant experiment between the two locales. Shaded red and blue areas represent the 95% CI.

Although the individuals used in the experiment showed no significant differences in maximum shell length between low and high intertidal plots prior to the transplant experiment (one‐way ANOVA: *F*_(1,55)_ = 2.263, *p* = 0.138), the morphometrics of *M. californianus* mussels differed depending on whether they were growing in the low or high intertidal. Individuals indigenous to the high intertidal had thicker shells (one‐way ANOVA: *F*_(1,55)_ = 66.28, *p* < 0.0001, Figure 3A), heavier shells (*F*_(1,55)_ = 64.17, *p* < 0.0001, Figure 3B), and higher total weights (*F*_(1,55)_ = 14.87, *p* = 0.0003, Figure 3C) compared to individuals indigenous to the low intertidal, while individuals indigenous to the low intertidal had wider shells compared to their high intertidal counterparts (*F*_(1,55)_ = 24.8, *p* < 0.0001, Figure 3D).

**FIGURE 4:**
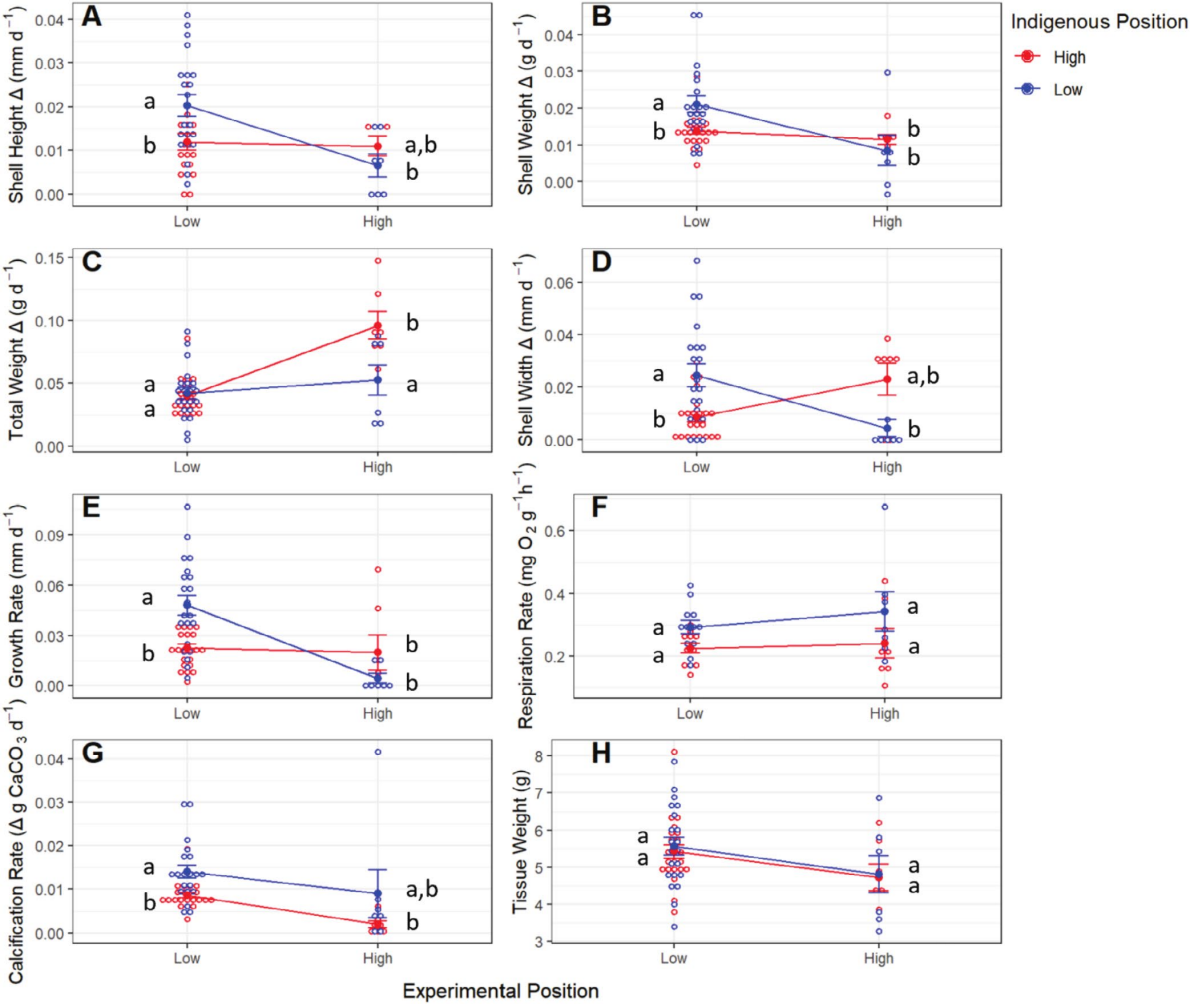
(A) Shell height change (mm day^−1^), (B) shell weight change (g day^−1^), (C) total weight change measured as weight in air (g day^−1^), (D) shell width change (mm day^−1^), (E) growth rate measured as change in shell length (mm day^−1^), (F) respiration rate measured as oxygen consumption (mg O_2_ g^−1^ h^−1^), (G) net calcification rate measured as change in buoyancy (g CaCO_3_ day^−1^), and (H) tissue weight (g) of in situ and transplanted *Mytilus californianus* individuals positioned in the low or high intertidal throughout the reciprocal transplant experiment. Individual data points and means ± SE are shown. Different letters beside each symbol indicate significant differences between experimental treatments evaluated using the Tukey HSD test as a post hoc comparison.

At the conclusion of the reciprocal transplant experiment, mussels from both plots showed a significant interaction between indigenous and experimental position in shell height (Figure 4A and Table 1A), shell weight (Figure 4B and Table 1B), total weight (Figure 4C and Table 1C), shell width (Figure 4D and Table 1D), and growth rate (Figure 4E and Table 1E). The shell height of in situ individuals in the high intertidal plot (mean = 0.010 ± SE = 0.002 mm day^−1^) had similar responses to transplanted mussels indigenous to the low intertidal (0.006 ± 0.002 mm day^−1^; Tukey HSD test: *p* = 0.8206, Figure 4A). However, in situ mussels in the low intertidal plot added more shell height (0.020 ± 0.002 mm day^−1^) when compared to mussels transplanted to the low intertidal plot (0.011 ± 0.001 mm day^−1^; Tukey HSD test: *p* = 0.0298, Figure 4A) and mussels transplanted to the high intertidal plot but indigenous to the low intertidal (0.006 ± 0.002 mm day^−1^; Tukey HSD test: *p* = 0.0089, Figure 4A). The weight of individual shells showed a similar response as in situ mussels in the high intertidal plot (0.011 ± 0.001 g day^−1^) as changes in shell weight did not significantly differ from transplanted mussels indigenous to the low intertidal (0.008 ± 0.004 g day^−1^; Tukey HSD test: *p* = 0.8926, Figure 4B). Additionally, in situ mussels in the low intertidal plot significantly increased their shell weight (0.021 ± 0.002 g day^−1^) when compared to those sourced from the high intertidal (0.013 ± 0.0009 g day^−1^; Tukey HSD test: *p* = 0.0192, Figure 4B) and to mussels transplanted to the high intertidal plot but indigenous to the low intertidal (0.008 ± 0.004 g day^−1^; Tukey HSD test: *p* = 0.003, Figure 4B).

**TABLE 1:** ANOVA results for mussel responses to a reciprocal transplant.

Biological responses	Source	df	MS	*F*	*p*
(A) Shell height ∆ (mm day^−1^)	Indigenous position (IP)	1	0.0003	4.113	**0.0476**
	Experimental position (EP)	1	0.0005	6.259	**0.0155**
	IP × EP	1	0.0004	4.72	**0.0343**
	Residuals	53	0.0001		
	Total	56			
(B) Shell weight ∆ (g day^−1^)	IP	1	0.0003	5.051	**0.0288**
	EP	1	0.0005	9.516	**0.0032**
	IP × EP	1	0.0002	4.485	**0.0389**
	Residuals	53	0.0001		
	Total	56			
(C) Total weight ∆ (g day^−1^)	IP	1	0.0011	2.385	0.1285
	EP	1	0.0118	25.676	**< 0.0001**
	IP × EP	1	0.0055	12.034	**0.0011**
	Residuals	53	0.0005		
	Total	56			
(D) Shell width ∆ (mm day^−1^)	IP	1	0.0008	3.554	0.0649
	EP	1	0.0001	0.371	0.5453
	IP × EP	1	0.0031	14.722	**0.0003**
	Residuals	53	0.0002		
	Total	56			
(E) Growth rate (mm day^−1^)	IP	1	0.0033	7.829	**0.0072**
	EP	1	0.0056	13.324	**0.0006**
	IP × EP	1	0.0044	10.46	**0.0021**
	Residuals	53	0.0004		
	Total	56			
(F) Respiration rate (mg O_2_ g^−1^ h^−1^)	IP	1	0.061	5.953	**0.0201**
	EP	1	0.0096	0.934	0.3406
	IP × EP	1	0.0028	0.273	0.605
	Residuals	34	0.0103		
	Total	37			
(G) Net calcification rate (g CaCO_3_ day^−1^)	IP	1	0.0005	10.545	**0.002**
	EP	1	0.0004	7.983	**0.0066**
	IP × EP	1	0.0001	0.145	0.7045
	Residuals	53	0.0001		
	Total	56			
(H) Tissue weight (g)	IP	1	0.209	0.195	0.6609
	EP	1	5.574	5.192	**0.0268**
	IP × EP	1	0.008	0.007	0.9337
	Residuals	53	1.074		
	Total	56			

*Note:* Effects on (A) shell height (mm day^−1^), (B) shell weight estimated as the buoyant weight (g day^−1^), (C) total weight measured as weight in air (g day^−1^), (D) shell width (mm day^−1^), (E) shell length (mm day^−1^), (F) respiration rate measured as oxygen consumption (mg O_2_ g^−1^ h^−1^), (G) net calcification rate measured as change in buoyancy (g CaCO_3_ day^−1^), and (H) tissue weight (g) in in situ and transplanted *Mytilus californianus* individuals positioned in the low or high intertidal throughout a reciprocal transplant experiment. Bold text shows significant *p*‐values at *α* = 0.05. Data are plotted in Figure 4.

In situ mussels in the high intertidal plot significantly increased their total weight (0.096 ± 0.011 g day^−1^) when compared to transplanted mussels indigenous to the low intertidal (0.052 ± 0.011 g day^−1^; Tukey HSD test: *p* = 0.002, Figure 4C). However, mussels transplanted to the low intertidal experienced significantly decreased weights (0.039 ± 0.002 g d^−1^) when compared to in situ mussels positioned in the high intertidal (0.096 ± 0.011 g day^−1^; Tukey HSD test: *p* = 9.0e‐7, Figure 4C). Total weight changes did not significantly differ when comparing in situ mussels indigenous to the low intertidal (0.042 ± 0.004 g day^−1^) to transplanted mussels indigenous to the high intertidal (0.052 ± 0.011 g day^−1^; Tukey HSD test: *p* = 0.9869, Figure 4C). Shell width changes were similar where in the high intertidal plot in situ mussels had increased shell width changes (0.023 ± 0.006 mm day^−1^) when compared to transplanted mussels indigenous to the low intertidal, although it was not significant (0.004 ± 0.003 mm day^−1^; Tukey HSD test: *p* = 0.090, Figure 4D). However, in the low intertidal plot, in situ mussels had significantly increased shell weight changes (0.024 ± 0.004 mm day^−1^) when compared to transplanted mussels indigenous to the high intertidal (0.008 ± 0.001 mm day^−1^; Tukey HSD test: *p* = 0.0044, Figure 4D), but did not significantly differ from in situ mussels indigenous to the high intertidal (0.023 ± 0.006 mm day^−1^; Tukey HSD test: *p* = 0.996, Figure 4D).

Growth rates, measured as change in shell length, of in situ low mussels in their indigenous position (0.047 ± 0.005 mm day^−1^) were significantly higher than mussels indigenous to the high intertidal for both treatments (in situ: 0.019 ± 0.010 mm day^−1^; Tukey HSD test: *p* = 0.013, transplant: 0.022 ± 0.002 mm day^−1^; Tukey HSD test: *p* = 0.0008, Figure 4E). However, in the high intertidal plot in situ mussels had higher growth rates than transplanted mussels indigenous to the low intertidal (0.019 ± 0.010 vs. 0.004 ± 0.002 mm day^−1^, Figure 4E) indicated by the significant interaction between indigenous and experimental position on the growth rates of individual mussels (Table 1E).

In their indigenous position, mussels from the low intertidal had significantly higher metabolic rates (0.292 ± 0.016 mg O_2_ g^−1^ h^−1^), measured as oxygen consumption, compared to high intertidal mussels (0.240 ± 0.046 mg O_2_ g^−1^ h^−1^; One‐way ANOVA: *F*_(1,35)_ = 6.087, *p* = 0.0185, Figure 4F). Experimentally transplanting mussels had no significant effect on oxygen consumption rates for mussels (Table 1F); transplanted mussels from the low intertidal maintained higher metabolic rates than mussels from the high intertidal regardless of where they were transplanted (0.342 ± 0.062 vs. 0.240 ± 0.046 mg O_2_ g^−1^ h^−1^, Figure 4F) and mussels indigenous to the high intertidal maintained lower metabolic rates regardless of their position (0.225 ± 0.01 vs. 0.292 ± 0.016 mg O_2_ g^−1^ h^−1^, Figure 4F). Metabolic rate assays from in situ neighboring individuals not used in the reciprocal transplant experiment confirmed these results, as low individuals maintained higher metabolic rates (0.374 ± 0.026 mg O_2_ g^−1^ h^−1^) compared to high intertidal individuals in common garden experiments (0.234 ± 0.022 mg O_2_ g^−1^ h^−1^; One‐way ANOVA: *F*_(1,30)_ = 16.8, *p* = 0.0003). Additionally, there was no significant difference in metabolic rates between experimental and non‐experimental individuals, suggesting that the transplantation itself did not significantly impact mussel function (Mann–Whitney *U* test: *W* = 716, *p* = 0.205).

Net calcification rates in mussels, measured as change in buoyancy, responded similarly to metabolic rates, where mussels indigenous to the low intertidal maintained higher net calcification rates than both transplanted (0.014 ± 0.001 vs. 0.008 ± 0.0006 g CaCO_3_ day^−1^, Figure 4G) and in situ (0.009 ± 0.005 vs. 0.001 ± 0.0008 g CaCO_3_ day^−1^, Figure 4G) mussels indigenous to the high intertidal. However, experimental position was shown to have a significant effect (Table 1G) where mussels positioned in the low intertidal had net calcification rates that were two times greater than mussels positioned in the high intertidal (0.011 ± 0.0008 vs. 0.005 ± 0.0028 g CaCO_3_ day^−1^), likely due to the longer periods of time spent underwater to feed and biomineralize CaCO_3_.

**FIGURE 5:**
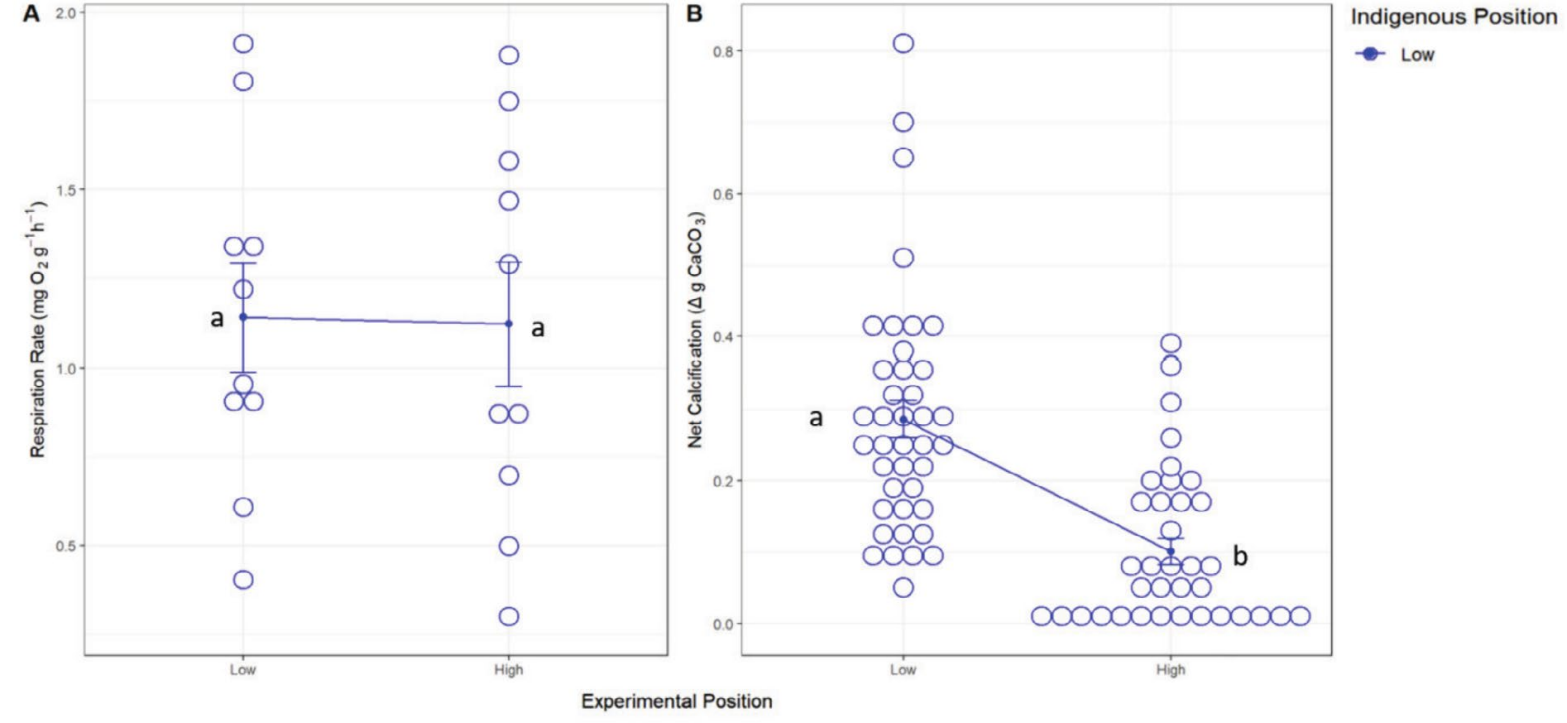
(A) Juvenile respiration rate measured as oxygen consumption (m O_2_ g^−1^ day^−1^) and (B) net calcification measured as total change in buoyancy (g CaCO_3_) of in‐situ and transplanted juvenile *Mytilus californianus* individuals positioned in the low or high intertidal throughout the reciprocal transplant experiment. Individual data points and means ± SE are shown. Different letters beside each symbol indicate significant differences between experimental treatments evaluated using the Tukey HSD test as a post hoc comparison.

For juvenile mussels, oxygen consumption rates at these smaller life stages were similar to adult mussels, where experimental position again did not significantly affect metabolic rate (Figure 5A and Table 2A), though juvenile mussels did have significantly higher metabolic rates (1.130 ± 0.112 mg O_2_ g^−1^ h^−1^) when compared to adult mussels (0.270 ± 0.017 mg O_2_ g^−1^ h^−1^; One‐way ANOVA: *F*_(1,55)_ = 103.8, *p* < 0.0001). Juvenile mussels also exhibited net calcification totals that were two times greater in the low intertidal than the high intertidal (0.286 ± 0.026 vs. 0.100 ± 0.018 g CaCO_3_, Figure 5B and Table 2B); however, adult mussels had significantly higher net calcification rates (0.009 ± 0.001 g CaCO_3_ day^−1^) when compared to juvenile mussels (0.003 ± 0.0003 CaCO_3_ day^−1^; one‐way ANOVA: *F*_(1,55)_ = 11.73, *p* = 0.0012).

**TABLE 2:** ANOVA results for juvenile mussel responses to a transplant.

Biological responses	Source	df	MS	*F*	*p*
(A) Respiration rate (mg O_2_ g^−1^ h^−1^)	Experimental position (EP)	1	0.0018	0.007	0.936
	Residuals	18	0.2665		
	Total	19			
(B) Net calcification (g CaCO_3_)	EP	1	0.6516	31.95	**< 0.0001**
	Residuals	74	0.0204		
	Total	75			

*Note:* Effects on respiration rate measured as (A) oxygen consumption (mg O_2_ g^−1^ h^−1^) and (B) net calcification total measured as change in buoyancy (g CaCO_3_) in in situ and transplanted *Mytilus californianus* (juvenile) individuals positioned in the low or high intertidal throughout a transplant experiment. Bold text shows significant *p*‐values at *α* = 0.05. Data are plotted in Figure 5.

Tissue weights in *M. californianus* individuals were lower for both transplanted and in situ mussels positioned in the high intertidal (4.76 ± 0.30 g) when compared to transplanted and in situ mussels positioned in the low intertidal (5.48 ± 0.151 g, Figure 4H and Table 1H), while indigenous position did not significantly affect tissue mass (Figure 4H and Table 1H). The long‐term transplant experiment with juvenile mussels showed even greater differences in tissue mass between mussels positioned in the low or high intertidal; low intertidal individuals maintained significantly higher tissue weights (4.14 ± 0.15 g) when compared to high intertidal individuals (1.84 ± 0.08 g; one‐way ANOVA: *F*_(1,53)_ = 99.25, *p* < 0.0001).

**FIGURE 6:**
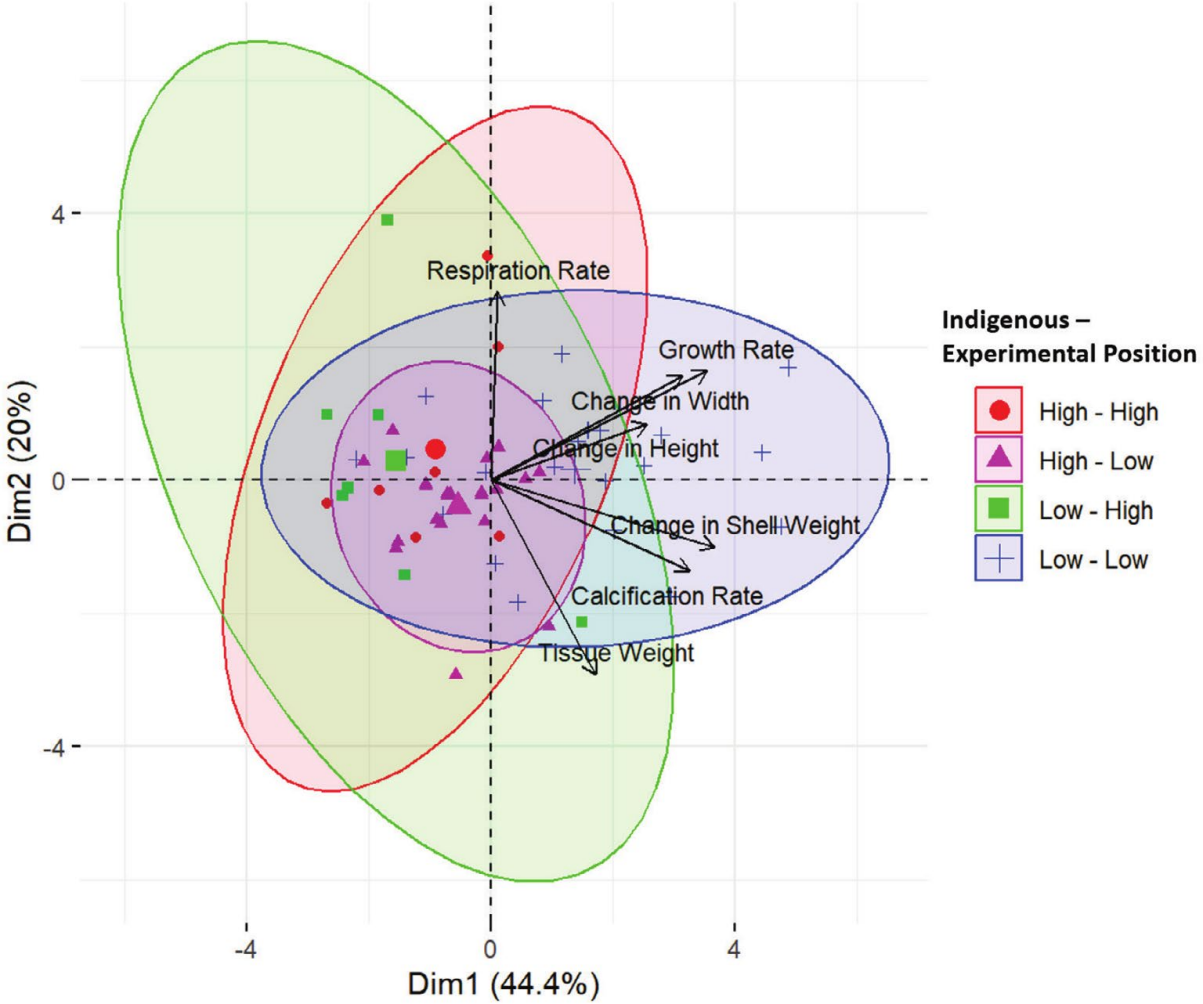
Biplot of principal component analysis (PCA) showing changes in phenotypic traits based on treatment for adult *Mytilus californianus* individuals throughout a reciprocal transplant experiment.

Visualizing all trait parameters from the reciprocal transplant experiment with PCA further demonstrated the dual influence the environment and indigenous position had on trait and morphological variation in mussels as phenotypic variation significantly differed among treatments (PERMANOVA: *F*_(3,53)_ = 6.289, *R* ^2^ = 0.26, *p* = 0.001, Figure 6). PC1 described 44.4% of the variation and was positively correlated with calcification rate, growth rate, and change in shell width, height, and weight. PC2 described 20% of the variation and was positively correlated with respiration rate and negatively correlated with tissue weight. The PCA plot portrays that individuals indigenous to the low intertidal were strongly correlated to traits associated with PC1, while in situ individuals indigenous to the high intertidal were strongly correlated to traits associated with PC2. Transplanted individuals indigenous to the low intertidal differentiated from low in situ individuals by showing stronger correlations to PC2 corresponding to the decreases in tissue weight (Figure 4H) that were observed for those individuals throughout the experimental period. Alternatively, transplanted individuals indigenous to the high intertidal differentiated from high in situ individuals by showing stronger correlations to PC1 corresponding to the increased net calcification rate that were observed for those individuals throughout the experimental period (Figure 4G).

### 3.3 Age Variation Within Populations

The age composition of the low and high intertidal mussels 39–51 mm in length ranged from 2 to 7 years of age. However, mussels from the high intertidal were significantly older, ranging from 4 to 7 with a mean of 5 years of age, compared to 3 years of age for low intertidal individuals (Students *T*‐test: *t*_(44)_ = 12.2, *p* < 0.0001). Additionally, mussels indigenous to the high intertidal showed higher age variability with a coefficient of variation of 0.158 compared to low intertidal mussels with a coefficient of variation of 0.151 (*F* test: *F*_(low df: 27,high df: 28)_ = 0.3145, *p* = 0.0035).

### 3.4 Spatially Varying Selection on Micro‐Geographic Scales

Analysis of the 638 candidate SNVs revealed high genetic differentiation with an average *F*_ST_ of 0.20 among the five populations. The experimental population on Tatoosh Island had a lower mean observed heterozygosity (*H*_O_ = 0.26) across both low and high intertidal individuals compared to the four inland populations (*H*_O_ = 0.40) and the expected heterozygosity (*H*_E_ = 0.44), signifying a reduction in genetic variability within the Tatoosh Island population. Furthermore, the Tatoosh Island population had a higher proportion of candidate SNVs compared to the inland populations (*P*_O_: 0.47 vs. 0.44). Of the 638 candidate SNVs, 222 were non‐synonymous mutations. Annotation of the 222 non‐synonymous variants using eggNOG‐mapper 2.1.12 uncovered multiple functional genes that have been characterized as affecting fitness across many bivalve species. These include genes involved in osmotic stress: VWDE (Abo‐Al‐Ela and Faggio 2021); oxidative stress: TXR (Wang et al. 2013); immune response: ubiquitin–protein, BIRC2 (Place et al. 2012; Nie et al. 2020); antioxidant defense: GPX3 (Hlaing et al. 2020); DNA repair and recombination: RAD54 (Connor and Gracey 2020); biomineralization: CA14 (Malachowicz and Wenne 2019); and heat stress: HSP70 (Place et al. 2012; Liu et al. 2014; Kourtidis et al. 2006; Nielsen et al. 2021; Hofmann and Somero 1995).

## 4 Discussion

### 4.1 Evidence for Phenotypic Plasticity

Environmental and possible genetic influences on phenotypic variation in the intertidal mussel *M. californianus* were revealed by the reciprocal transplant and transplant experiments, demonstrating that environmental factors vary spatially, and result in spatially varying differentiation. The significant interaction between indigenous and experimental position on morphological traits such as shell length, shell height, shell weight, total weight, and shell width (Table 1), suggest a genotype × environment interaction generates phenotypic variation among these traits in *M. californianus* mussels. Indigenous and experimental position also significantly affected net calcification rates for both adult and juvenile mussels; however, the lack of a significant interaction between indigenous and experimental position (Tables 1 and 2) suggests both genotype and environment affect calcification but not an interaction between the two. Alternatively, for tissue weights only the environment had a significant effect (Table 1H), while indigenous position and genotype were not significant. Thus, we can conclude that phenotypic differences in shell morphology, calcification, and tissue weight are a result of phenotypic plasticity with genotype or canalization having an additional effect on shell morphology and calcification but no effect on tissue weight. Given that heat stress and limited food availability has been shown to decrease tissue weight in *M. californianus* individuals (Fitzgerald‐Dehoog et al. 2012), the decreases in tissue weights for animals that were positioned in the high intertidal are most likely a cost to being in an environment that experiences prolonged heat and aerial exposure events, whereas morphological plasticity is a tradeoff that allows these animals to maximize fitness in different environments. These results demonstrate that morphological traits for populations who range across heterogenous environments may exhibit phenotypic plasticity over fixed phenotypes or local adaptation.

### 4.2 Evidence for Local Adaptation

Local adaptation is advantageous when phenotypic plasticity, which allows an organism to adjust to varying environments, is costly and constrains the fitness of a single (plastic) genotype across diverse conditions, resulting in distinct genetic variants adapted to specific environments within a population (Yeaman 2022; Tienderen 1997). As selective pressures intensify, divergent selection enhances genetic differentiation by counteracting the neutral effects of gene flow, thereby promoting local adaptation and reducing plasticity (Wright 1969; Wadgymar et al. 2022). In the case of *M. californianus* , the metabolic rates of both adult and juvenile mussels were unrelated to where they were transplanted. Instead, mussels retained the features of their indigenous position (Tables 1 and 2), indicating that oxygen consumption in mussels is locally canalized or adapted to the specific conditions of their native environment and is not a plastic response to environmental conditions. Notably, mussels from high intertidal zones, which experience greater heat and aerial exposure stress, maintained lower metabolic rates compared to those from low intertidal, even when they were transplanted to the more favorable low intertidal (Figure 4F).

Analyses of the DNA sequences of low and high intertidal mussels revealed further evidence of local adaptation; individuals indigenous to the high intertidal had increased frequencies of non‐synonymous mutations in HSP70, a protein known to be differentially expressed between high and low *M. californianus* mussels (Place et al. 2012) and shown to significantly affect fitness across all six *Mytilus* spp. (e.g., Liu et al. 2014; Kourtidis et al. 2006; Nielsen et al. 2021; Hofmann and Somero 1995; Roberts et al. 1997; Osores et al. 2017). Individuals indigenous to the low intertidal also had increased frequencies of non‐synonymous mutations in CA14, a protein involved in calcification (Malachowicz and Wenne 2019). Non‐synonymous mutations can be an indicator of adaptive evolution (McDonald and Kreitman 1991), and increased allele frequencies are typically linked to adaptive genetic differentiation (Jump et al. 2006; Liggins et al. 2020) that leads to a reduction in genetic variability (Bulmer 1971). Here, whole genome sequencing (WGS) suggests that high intertidal individuals experience increased selection in HSP70, whereas low intertidal individuals experience increased selection in CA14. Our demonstration that in situ individuals indigenous to the high intertidal had increased growth rates compared to transplanted individuals (Figure 4E), while individuals indigenous to the low intertidal had increased calcification rates compared to individuals indigenous to the high intertidal (Figure 4G), indicates that particular proteins may enable organisms to thrive in their specific environmental niches.

### 4.3 Genetic Consequences of Adaptive Traits

Quantifying phenotypic change across a population that spans a strong ecological gradient reveals the patterns and processes driving adaptive trait evolution. For example, if environmental and genetic influences on phenotypic variation act in concert (selection and plasticity covary positively), phenotypic differences will be distinct among native environments, and the phenotype of transplanted individuals would converge toward the indigenous phenotype, a phenomenon termed cogradient variation (CoGV) (Trussell 2000). In contrast, if environmental and genetic influences on phenotypic variation act in opposition (selection and plasticity covary negatively), there is little phenotypic variation among native individuals, and transplanted individuals would be distinct in their phenotype from the native phenotype in the same environment, known as countergradient variation (CnGV) (Levins 1968). The effects of CoGV and CnGV on functional trait evolution has revealed that CoGV traits more often involve morphology, whereas CnGV traits primarily involve physiological characteristics (Marcil et al. 2006; Conover et al. 2009). Our results support these findings as morphological traits in *M. californianus* mussels demonstrated greater phenotypic plasticity than physiological traits such as metabolic rate. Here, as in other studies within species (Marcil et al. 2006; Conover et al. 2009), we can expect functional differences in how traits evolve in response to environmental change.

Evolutionary changes in species traits are dependent on the rate and direction of environmental change and existing genetic variation within plastic or locally adapted phenotypes. In changing environments, adaptive phenotypic plasticity can often ameliorate extinction by allowing time for populations to respond to new conditions (Kelly 2019). Additionally, genetic variation within plastic traits can lead to selection for plasticity, resulting in increased plasticity toward new fitness optima, known as genetic accommodation (Lande 2009; Baldwin 1896; Scheiner et al. 2017). However, if the magnitude of change across a species range exceeds the thresholds of plasticity or plasticity decreases organismal fitness in their new environment (i.e., maladaptive plasticity) (Stamp and Hadfield 2020), an adaptive phenotypic response can be delayed, and the chance of extinction increased (Scheiner et al. 2017). In cases such as this where environmental change is large, locally adapted traits that are tightly linked and characterized by coadapted gene complexes are selected as they can be inherited in a single generation (Yeaman 2022). This results in the rapid adaptation of populations as coadapted gene complexes selectively sweep through a population (i.e., soft sweep) which decreases the likelihood of extinction (Messer and Petrov 2013) and increases organismal fitness in the new environment. However, in the long term, soft sweeps could be disadvantageous for organisms who persist across rapidly fluctuating environments, such as the marine intertidal, as it reduces genetic variability and phenotypic plasticity, thereby reducing organismal response to diverse conditions (Lalejini et al. 2021). The interplay between genetic variation and environmental change on adaptive traits is crucial for the survival of populations in changing ecosystems. While adaptive phenotypic plasticity and the rapid adaptation of locally adapted traits can mitigate extinction risks, the potential drawbacks, such as maladaptive plasticity and reduced genetic diversity, underscore the complexities of evolutionary responses in dynamic environments. Understanding the genetic consequences increasing selective pressures have on different functional traits across ecological gradients is essential for predicting how species will cope with ongoing environmental challenges and will assist resource managers to proactively plan for these changes.

### 4.4 Drivers of Microgeographic Differentiation

The selective pressures associated with emersion stress within the marine intertidal are repeatedly associated with intraspecific differentiation on microgeographic scales (< 10 m) and are likely a significant driver of genetic and phenotypic divergence in intertidal marine species (Hays et al. 2021). This phenomenon arises partly from the combined effects of daily tidal fluctuations and coastal elevational gradients, which create daily temperature ranges comparable to those observed across extensive latitudinal gradients that exceed 1000 km, yet occur on scales of less than 10 m (Denny et al. 2011; Helmuth et al. 2006). Abiotic forces in the marine intertidal lead to divergence in *M. californianus* mussel beds along elevational gradients, as demonstrated by thicker shells (Figure 3A) and increased periostracum loss from increased incidence of endolithic phototroph infestation (Figure 2) (Gehman and Harley 2019). While endolithic phototroph infestation is often lethal in *Mytilus* spp. because it accelerates shell degradation rates (Marquet et al. 2013), it can be beneficial for high intertidal individuals as the loss of periostracum whitens the individual's shell, significantly increasing reflectivity and decreasing body temperatures during extreme heat stress, thereby reducing mortality (Gehman and Harley 2019; Zardi et al. 2016).

Abiotic factors similarly shape elevational gradients and drive species differentiation in terrestrial systems as well. In mountainous regions, higher elevations are linked to lower temperatures, increased soil moisture, and shorter growing seasons (Dunne et al. 2003; Anderson and Gezon 2015), leading to microgeographic adaptations in various species. Examples include the perennial herb *Boechera stricta* (Anderson et al. 2015), the deciduous tree *Fagus sylvatica* (Gauzere et al. 2020), and insects such as *Dichroplus pratensis* and *D. vittatus* (Bidau et al. 2012). In both marine and terrestrial systems, strong environmental gradients generate selective pressures strong enough to override the neutralizing effects of gene flow, leading to microgeographic adaptation. These extreme gradients contribute to genetic and phenotypic divergence in species, revealing how the intensity of environmental stressors on small spatial scales contributes to the evolution and adaptation of species who persist across heterogeneous environments.

## 5 Conclusion

The complex relationship between genetic and environmental influences on phenotypic variation presents a major challenge to understanding the consequences of global change for species. In this study, we provided empirical evidence of spatially varying selection driving trait differentiation within the marine intertidal showing that adaptive trait evolution differs between functional traits, and that future evolutionary responses will be determined by the magnitude of environmental change and existing genetic adaptations (Burrows et al. 2011; Sunday et al. 2011). For foundation species, these responses will undoubtably reverberate through communities and alter ecosystem function (Des Roches et al. 2018; Whitham et al. 2020; Smee et al. 2013). Although there is evidence of the community and ecosystem implications of intrapopulation variation in foundational species (Hersch‐Green et al. 2011), our understanding of the microevolutionary processes responsible for creating and sustaining within‐species genetic diversity remains relatively limited. Our experiments revealed high intertidal mussels, who persist in an extreme environment, had significantly lower metabolic rates, greater age variability, and increased frequencies of non‐synonymous mutations in functionally relevant genes like HSP70 compared to their low intertidal counterparts, suggesting intrapopulation differentiation may play a key role in species persistence in stressful environments. As global change intensifies stress gradients across species ranges, understanding how these evolutionary responses feedback into phenotypic response and other ecological processes will be imperative for predicting future ecosystem function.

## Author Contributions

**Casey S. Richards:** conceptualization (lead), formal analysis (lead), investigation (lead), writing – original draft (lead). **Diana Vera Cruz:** data curation (equal), formal analysis (equal), software (lead), writing – review and editing (equal). **Jason W. Shapiro:** data curation (equal), formal analysis (equal), software (lead), writing – review and editing (equal). **J. Timothy Wootton:** conceptualization (supporting), writing – review and editing (equal). **Catherine A. Pfister:** conceptualization (supporting), supervision (lead), writing – review and editing (lead).

## Conflicts of Interest

The authors declare no conflicts of interest.

## Data Availability

Genetic data deposited to the National Center for Biotechnology Information's (NCBI) Sequence Read Archive (BioProject ID: PRJNA1245383). Data and code used for analyses are made available on Dryad: https://doi.org/10.5061/dryad.pnvx0k70n.
